# Supplementary material for: Multiple Facets of Nitrogen: From Atmospheric Gas to Indispensable Agricultural Input
Source: Life (Basel). 2022 Aug 19;12(8):1272. doi: 10.3390/life12081272 (PMC9410007; doi:10.3390/life12081272)
Supplement: Supplementary file 1 [file life-12-01272-s001.zip › life-1841899-supplementary.pdf]

Table S1. Effects of secondary macronutrients and micronutrients deficiency and excess.

Plant Nutrients	Function	Deficiency Symptoms	Excess/Toxicity
Secondary nutrients (Ca, Mg, and S)			
Calcium (Ca)	- Component of cell walls in the form of calcium-pectate, necessary for normal mitosis or cell division.<br>- Helps in membrane stability, maintenance of-chromosome structure.<br>- Activator of enzymes, including pholipase, argine kinase, adenosine triphosphates).<br>- Acts as a detoxifying agent by neutralizing-organic acids in plants.<br>- Acts a signaling molecule and interacts with other-signaling pathways as part of the adaptive response mechanims towards stress tolerance	Calcium deficiencies are not often seen in the field because secondary effects associated with high acidity limit growth.<br>Failure in the development of terminal bud, dead spots in the mid rib of some plants. In corn, tip of the new leaves may be covered with a sticky, gelantinous material that causes them to adhere to one another<br>The young leaves of new plants are affected first. These are often distorted, small and abnormally dark green.<br>Leaves may be cup-shaped and crinkled and the terminal buds deteriorate with some breakdown of petioles.<br>- Root growth is markedly impaired, rotting of roots occurs.<br>- Desiccation of growign points (terminal buds) of plants under severe deficiency.<br>- Buds and blossoms shed prematurely.<br>- Stem structure weakened.	- High calcium can cause magnesium or boron deficiencies
Magnesium (Mg)	- Component of chlorophyll molecule and therefore essential for photosynthesis (or food synthesis in plants)<br>- An activator of many enzyme systems involved in carbohydrate metabolism, syntehsis of nucleic acids, etc.<br>- Promotes uptake and translocation of phosphorus.<br>- Helps in movement of sugars within the plant.	- Light green leaves and yellowing of leaves similar to nitrogen deficiency.<br>- Interveinal chlorosis, mainly of older leaves, producing a streaked or patchy effect; with acute deficiency, the affected tissue may dry up and die.<br>- In some vegetable plants, chlorotic spots between veins, and marbling with tints of orange, red and purple.<br>- Twigs weak and prone to fungus attack, usually premature leaf drop.	- High magnesium can cause calcium deficiency

Sulfur (S)	- Constituent of sulphurbearing amino acids.<br>- Involved in the metabolic activities of vitamins, biotin, thiamine and coenzyme A.<br>- Aids stabilization of protein structure.	- Younger leaves turn uniformaly yellowish green or chlorotic.<br>- Shoot growth is restricted, flower production often indeterinate.<br>- Stems are stiff, woody and small in diameter.
Micronutrients (Fe^{2+}, Zn^{2+}, Mn^{2+}, Cu^{2+}, BO_3^-, MoO_4^-, and Cl^-)		
Zinc (Zn)	- Involved in the biosynthesis of indoe acetic acid.<br>- Essential component of a variety of metallo-enzymes – carbonic anhydrase, alcohol dehydrogenase, etc.<br>- Plays a role in nucleic acid and protein synthesis.<br>- Regulate transption of specific genes<br>- Assists the utilization of phosphorus and nitrogen in plants.<br>- Formation of auxins and chloroplast; carbohydrates metabolism, stabilizing and structural orientation of membrane proteins.	- Deficiency symptoms mostly appear on the second or third fully expanded leaves from the top of the plant.<br>- Stunted growth, pale to white coloration of young leaves, white bud and white streaks in leavs of corn; brownish red (rusty) discoloration of leaves in rice commonly known as Khaira disease in rice. Corn, beans citrus, and rice are indicator plants for zinc deficiency.<br>- In maize, from light yellow striping ot a broad band of white or yellow tissue with reddish purple veins between the midrib and edges of the leaf, occurring mainly in the lower half of the leaf.<br>- In wheat, a longitudinal band of white or yellow leaf tissue, followed by interveinal chlorotic mottling and white to brown necrotic lesions in the middle of the leaf balde, eventual collapse of the affected leaves near the middle.<br>- In rice, after 15–20 days of transplanting, small scattered light yellow spots appear on the older leaves which later enlarge, coalesce and turn deep borwn; the entire leaf becomes rust-brown in color and dries out within a month.<br>- In citrus, irregular interveinal chlorosis, terminal leaves become small and narrowed (little-leaf), fruit-bud formation is severely reduced, twigs die back.
Copper (Cu)	- Constituent dof cytrochrome oxidase and component of many enzymes, including ascorbic acid oxidase, phenolase, lactase.	- In cereals, yellowing and curling of the leaf blade, restricted ear production and poor grain set, indeterminate tillering.

	- Promotes formation of vitamine A in plants.	- In citrus, dieback of new growth, exanthema pockets of gum develop between the bark and the wood, the fruit shows brown excrescences.	
Iron (Fe)	- Structural component of cytochromes, perrichrome, and leghemoglobin<br>- Is utilized as a co-factor in various enzymatic reactions, and is present in the form of heme and iron-sulfur (Fe-S) prtoeins that take part in photosynthesis (synthesis of chlorophyll), respiration, and assmilation of nitrogen and sulfur.<br>- Is engaged in the transfer of electrons in electron transport chain (ETC) and in many ubiquitous and other diverse metabolic and physiological processes such as germination, flower initiation, stomatal movement,etc.<br>- Necessary for the synthesis and maintenance of chlorophyll in plants.<br>- Essential component of many enzymes.<br>- Plays an essential role in nucleic acid metabolism, affects RNA metabolism or chloroplats.	- Typical interveinal chlorosis, yelowing or whitening of young leaves, youngest leaves are first affectedm points and margins of leaves keeps their green color longest.<br>- In severe deficiency, the entire leaf, veins and interveinal areas turn yellow, pale or white, or may eventually become bleached.<br>- Iron deficiency due to lack of its uptake or absorption results in low photosynthetic efficiency and reduced growth<br>- Severe losses in quality and crop yields.	- Excessive Fe availability in the soil results in the inhibition of plant growth and development<br>- Distrubance of basic plant metabolism due to disruption in the rate of uptake and translocation of other key nutrients.<br>- Excess Fe compete, in roots rhizosphere, for the same membrane-localized channels and transporter proteins, causes defiency in those nutrients
Manganese (Mn)	- A catalyst in several enzymatic and physiological reactions in plants.<br>- A constituent of pyruvate carboxylase.<br>- Involvedd in the resporatory process in plants.<br>- Activates enzymes concerned with the metabolism of nitrogen and synthesis of chlorophyll.<br>- Controls the reduction-oxidation potential in plant cells during the phases of light and darkness.	- Chlorosis between the veins of young leaves, characterized by the appearance of chlorotic and necrotic spots in the interveinal areas.<br>- Greyish areas appear near the base of the younger leavse and become yellowish to yellow orange.<br>- Symptoms of deficiency popularly known in oats as grey speck, in sugarcane as streak disease.	- Reduced growth rate and necrosis along the main veins.<br>- Toxicity symptoms start on the lower leaves and work up the main stem.<br>- The leaves die back to the stem.
Boron (B)	- Affects the activities of certain enzymes.	- Death of growing plants (shoot tips).	- Yellow-tinted band around the leaf margins.

	- Ability to complex with various polyhbydroxy-compounds.<br>- Increases permeability in membrance and thereby facilitates carbohydrates transport.<br>- Involved in lignin syntehsis and other reasctions.<br>- Essential for cell division.<br>- Associated with the uptake of calcium and its utilization by plants.<br>- Regulates potassium/calcium ratio in plants.<br>- Essential for protein synthesis.	- The leavs have a thick texture, sometimes curling and becoming brittle.<br>- Flowers do not form and roots growth is stunted.<br>- Brown heart in root crops characterized by dark spots on the thickest part of the root or splitting at center.<br>- Fruits such as apples develop internal and external cork symptoms.	- The chlorotic wone becomes necrotic and gray, while the major portion of the leaf remains green.
Molybdenum (Mo)	- Associated with nitrogen utilization and fixation.<br>- Constituent of nitrate reductase and nitrogenase.<br>- Required by *Rhizobia* for nitrogen fixation.	- Chlorotic interveinal mottling of the lower leaves, followed by marginal necrosis and infolding of the leaves.<br>- In cauliflower, the leaf tissues wither leaving only the midrib and a few smal pices of leaf blade (whiptail).<br>- Molybdenum deficiency is mardely evident in leguminous plants.	
Chlorine (Cl)	- A constituent of auxin-chloroindole-3-acetic acid, which in immature seeds takes the place of indoloe acetic acid.<br>- Constituent of many compounds found in fungi and bacteria.<br>- Stimulates the activity of some enzymes and influences carbohydrate metabolism and water holding capacity of plant tissues	- Wilting of leaflet tips, chlorosis of leaves and finally bronzing and drying.	

Table S2. Nutrient elements form taken up by plants.

Plant nutrients	Uptake form	Chemical form
	Primary nutrients	
Nitrogen	Nitrate and Ammonium	NO_3^- and NH_4^+
Phosphorus	HPO_4^- and $H_2PO_4^-$	HPO_4^- and $H_2PO_4^-$
Potassium	Potash	K_2O
	Secondary nutrients	
Calcium	Ca^{2+}	Ca^{2+}
Magnesium	Mg^{2+}	Mg^{2+}
Sulphur	Sulfate	SO_4^{2-}
Iron	Ferrous ion	Fe^{2+}, Fe^{3+}
Zinc	Zinc ion	Zn^{2+}
Manganese	Manganese ion	Mn^{2+}, Mn^{3+}
Copper	Copper ion	Cu^{2+}
Boron	Borate	BO_3^{2-}
Molybdenum	Molybdenate	MoO_4^{2-}
Chlorine	Chlorine ion	Cl^-
